# The Cerebral Localization of Pain: Anatomical and Functional Considerations for Targeted Electrical Therapies

**DOI:** 10.3390/jcm9061945

**Published:** 2020-06-22

**Authors:** Rose M. Caston, Elliot H. Smith, Tyler S. Davis, John D. Rolston

**Affiliations:** Department of Neurosurgery, University of Utah, Salt Lake City, UT 84106, USA; rose.caston@hsc.utah.edu (R.M.C.); e.h.smith@utah.edu (E.H.S.); tyler.davis@hsc.utah.edu (T.S.D.)

**Keywords:** deep brain stimulation, closed loop, sensing, electrophysiology, neurophysiology, pain, thermal grill, imaging

## Abstract

Millions of people in the United States are affected by chronic pain, and the financial cost of pain treatment is weighing on the healthcare system. In some cases, current pharmacological treatments may do more harm than good, as with the United States opioid crisis. Direct electrical stimulation of the brain is one potential non-pharmacological treatment with a long history of investigation. Yet brain stimulation has been far less successful than peripheral or spinal cord stimulation, perhaps because of our limited understanding of the neural circuits involved in pain perception. In this paper, we review the history of using electrical stimulation of the brain to treat pain, as well as contemporary studies identifying the structures involved in pain networks, such as the thalamus, insula, and anterior cingulate. We propose that the thermal grill illusion, an experimental pain model, can facilitate further investigation of these structures. Pairing this model with intracranial recording will provide insight toward disentangling the neural correlates from the described anatomic areas. Finally, the possibility of altering pain perception with brain stimulation in these regions could be highly informative for the development of novel brain stimulation therapies for chronic pain.

## 1. Introduction

Pain is a unique and unorthodox sense. We reflexively respond to pain without being conscious of it, yet the conscious experience of pain can be overwhelming, debilitating, and chronic, depending on the cognitive, emotional, and allostatic context. Although the spinal reflexes mediate the rapid, unconscious avoidance of nociceptive stimuli, the experience of pain relies on the central nervous system’s amplification and abstraction of nociception. In chronic pain, the healthy, adaptive relationship between experiential pain and nociception becomes disrupted. Pain-inducing contextual factors often replace pain-inducing nociception.

Two general categories of chronic hypersensitivity to pain exist. Allodynia is pain resulting from hypersensitivity to normal innocuous somatosensory (non-painful) stimuli. For example, pain resulting from brushing a hand with a feather [1]. Hyperalgesia is increased sensitivity, intensity, or duration of painful stimuli [1,2]. Both of these conditions can arise from hyperexcitability of the dorsal horn of the spinal cord (i.e., where pain fibers first synapse). However, it is thought these conditions may also be “centralized,” presumably via inappropriate neuronal computations in a circuit comprised of the anterior cingulate cortex (ACC), insular cortex, and thalamus. For example, a burn can transiently cause a reduction of the pain threshold for mechanical stress, but the cognitive and affective elements of a burn can induce chronic hyperalgesia. Allodynia and hyperalgesia occur with spontaneous pain in chronic pain disorders.

Chronic and acute pain have sparked a growing interest in recent years. Chronic pain is widespread. In 2008, an estimated 100 million adults in the United States (U.S.) were affected by chronic pain. Total health care expenses attributable to pain, along with the amount associated with lower worker productivity, cost the U.S. between $560 and $635 billion per year [3]. This estimate is higher than the annual cost of heart disease ($309 billion), cancer ($243 billion), or diabetes ($188 billion) [3,4]. Pain treatment is complex and multidimensional, often involving medical providers, pain clinics, and state governments regulating narcotic drugs [5]. Narcotics, specifically opioids, are a poor treatment solution for two significant reasons—tolerance and lethality. Opioid use is associated with tolerance, as larger doses are required to achieve the same level of pain relief, yet high doses of opioids can be lethal. In 2016, 64,000 people died from drug overdoses in the U.S.—42,000 of those deaths were opioid related [6]. Opioids are not a viable long-term treatment for chronic pain. One potential solution is to design less systemic, more targeted interventions to disrupt pain signals within the brain, for example with electrical stimulation.

The use of intracranial electrical stimulation began in the mid-1900s, with the development of stereotactic frames. These devices enabled early psychologists/physiologists/psychiatrists to explore the effects before lesioning the areas of interest for a therapeutic effect [7]. Heath, a psychiatrist [8], implanted electrodes in schizophrenic patients to study the effects of intracranial stimulation. He discovered that electrical stimulation resulted in euphoria and analgesia, especially with stimulation of the septal area [9,10,11,12,13,14]. In 1954, deep brain stimulation (DBS) rat experiments alleviated pain with stimulation targeted to the septal nuclei, mammillothalamic tract, and cingulate cortex [14,15,16]. After these promising animal studies, stimulation of the human septum (intending to treat pain) proved less successful, with only one out of six patients with terminal cancer experiencing reduced pain [16,17].

The motivation to treat pain using electrical stimulation increased with the introduction of Melzack and Wall’s gate theory in the 1960s [18,19]. Gate theory is the notion that non-painful inputs close nerve “gates” through the activation of Aβ fibers. Gate closure disrupts a painful input from traveling along signaling pathways to the central nervous system, a process that is the basis for spinal cord stimulation (SCS). SCS is currently approved by the U.S. Food and Drug Administration (FDA) to treat chronic pain of the trunk and limbs, intractable low back pain, leg pain, and pain from failed back surgery syndrome. In Europe, SCS is also approved for refractory angina pectoris and peripheral limb ischemia [20]. Success using SCS suggests that stimulation of other targets within the central nervous system may also modulate pain signaling. Positive results demonstrated by cortical stimulation support this. A recent pooled effect from 12 trials found motor cortex stimulation improves pain by 65.1% in postradicular plexopathy, 46.5% in trigeminal neuropathy, 35.2% after stroke, 34.1% in phantom limbs, and 29.8% in plexus avulsion [21]. Motor cortex stimulation works by affecting the activity in the thalamic nuclei and somatosensory regions. It modulates a vast network of structures, including the cerebellum, striatum, ventral posterolateral nucleus, and other thalamic areas. A more targeted approach is highly favorable to decrease the negative side effects associated with electrically stimulating all of these structures.

Whereas the path from Melzack and Wall’s gate theory to present-day SCS is well defined, the path of DBS through the late 1900s meanders. After the disappointing results of septal DBS, studies continued on a wide array of potential anatomical brain targets in rodents and humans throughout the mid-to-late 1900s [22,23,24,25,26,27,28]. When studies translated from animals to human studies, they varied in electrode numbers, stimulation parameters, and anatomical targets, leading to inconsistent conclusions [17]. The FDA requested industry intervention to provide further data on safety and efficacy [14,29]. The first Medtronic study failed to show that half the patients had at least 50% pain relief [17]. The second trial failed because of a lack of enrollment (further details provided in Section 2). In 1989, the FDA rejected using DBS for chronic pain treatment [29].

**Figure 1:**
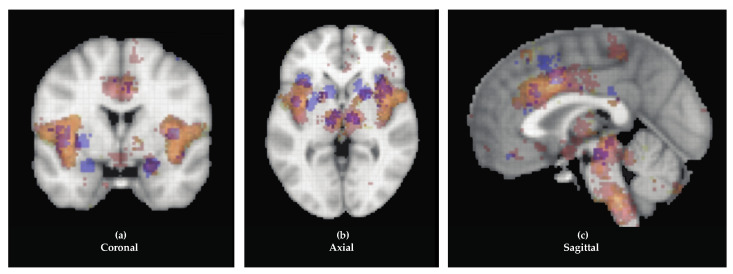
Overlay of three color-coded pain-related terms. “Chronic pain” is blue, “painful” is yellow, and “pain” is red. Functional magnetic resonance imaging (fMRI) studies of these terms are visualized in coronal (**a**), axial (**b**), and sagittal (**c**) axes from the Neurosynth database (neurosynth.org), showing consistent activation of the anterior cingulate cortex (ACC), thalamus, insula, and brainstem.

To design safe and effective targeted therapies, such as DBS, it is critical to understand more about the circuits involved in pain processing in time and space and the potentially distributed nature of pain encoding in the human brain. The peripheral and spinal circuitry involved in pain is well characterized [30]. Here, we instead focus on the cerebral localization of pain networks, with an eye towards the development of targeted neurostimulation therapies for chronic pain [17]. The structures discussed in this review—such as the somatosensory cortex, thalamus, insula, and the ACC—consistently correlate with painful stimuli [17,31,32,33,34]. Neuroimaging studies routinely demonstrate activation in these areas, as shown in Figure 1 [35]. Therefore, we discuss the processing of conscious perception of pain in the cerebrum, the historical electrical stimulation of these regions, if pertinent, and the future application of DBS to modulate this activity.

## 2. Anatomical Background

### 2.1. Somatosensory System and Relevant Inputs

Pain-sensing neurons, nociceptors, are pseudounipolar cells with somata, located in the dorsal root ganglion within the dorsal root of spinal nerves. Nociceptors were first described by Sherrington in 1906 [36]. Their afferent processes, either unmyelinated C fibers or myelinated Aδ fibers, project to peripheral tissues and typically end with free nerve endings capable of sensing painful mechanical or thermal stimuli. The efferent processes project to the thalamus, hypothalamus, and several midbrain areas, mainly via the contralateral anterolateral spinal cord. The disruption of information transmission along this tract via either surgical transection (first described by Spiller and Martin in 1912 [37]) or electrical stimulation has shown notable success in ameliorating some forms of chronic pain [38].

**Figure 2:**
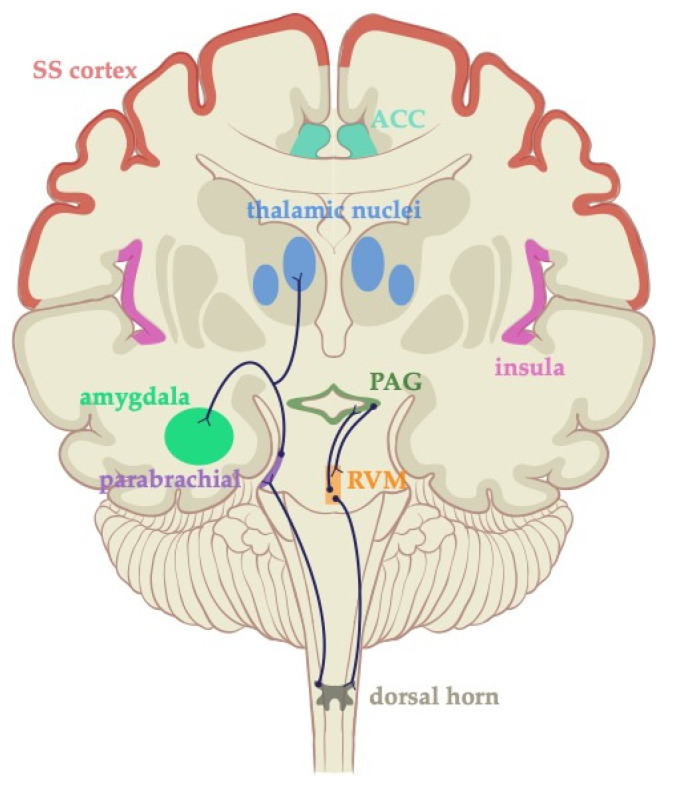
Descending pain modulatory pathway. The pathway originates in the cerebrum and descends to the periaqueductal gray (PAG) matter, rostral ventromedial medulla, and projects to the dorsal horn of the spinal cord. Bidirectional nociceptive pathways through the medullary nuclei are shown. The efferent pathway from the dorsal horn goes through the parabrachial nucleus, amygdala, and thalamic nuclei.

The descending pain modulatory pathway travels from the cerebrum to the ventrolateral periaqueductal gray (PAG) matter and rostral ventromedial medulla [39], and it projects to the dorsal horn of the spinal cord, as shown in Figure 2. The PAG matter in the brainstem contains bidirectional nociceptive pathways from medullary nuclei, allowing for modulation through both projections. As with other medullary nuclei, analgesia occurs via inhibitory serotonergic, enkephalin, mu-opioid, and GABA projections to the dorsal horn through descending pain control pathways. The other efferent nuclei from the medulla and pons appear to have a similar mechanism of analgesia. These are the same pathways thought to underlie opiate-induced analgesia [30]. Other notable efferents from the dorsal horn include the parabrachial nuclei, which project directly to the amygdala. The Kölliker-Fuse nucleus is found within the parabrachial nucleus and demonstrates noradrenergic descending inhibition [40]. Therefore, the parabrachial nuclei may be involved with salience or affective elements of pain [41,42].

Nociceptive projections ascend through the PAG and then project to the thalamic nuclei, which have medial and lateral nuclear groups [43]. The lateral group, consisting of ventroposterior nuclei, has small receptive fields and projects to the somatosensory cortex. Circuits involving these nuclei are therefore likely responsible for the conscious identification and localization of painful stimuli. The medial group consists of the central lateral nucleus and intralaminar complex, which have widespread projections to the basal ganglia and cortex, and therefore likely mediate the arousal response to pain.

Following the disappointing results of septal stimulation in the 1960s, electrical stimulation of the PAG was tried in humans in the 1970s [16,17]. In one of the earliest studies on PAG stimulation, Richardson and Akil [26] reported on 30 patients with electrodes implanted for patient-controlled self-stimulation. Two thirds of the patients reported good outcomes (reduction in pain of >50%). Periventricular stimulation showed similar promise around the same time, with Hosobuchi et al. [28] showing pain relief in five of six patients. Young et al. [44] showed that stimulation of the Kölliker-Fuse subnucleus alone or in combination with stimulation in the PAG matter or the somatosensory thalamic nuclei provided self-reported “excellent” pain relief in three of six patients with intractable pain. Following these, and other promising early studies, were two negative randomized controlled trials involving Medtronic devices. One trial, based on the Model 3380 electrode, was discontinued after the first-generation production. The other trial involved the Model 3387 electrode. This clinical trial was stopped because of slow enrollment, high attrition, and low efficacy [29].

### 2.2. Thalamus

While the thalamus is like a relay station for nociceptive information, it also likely performs important computations on afferent nociceptive information. The most obvious way this could occur is by regulating cortical excitability via its activity or connectivity. Thalamic lesions in experimental animals seem to support this role, causing moderate changes in pain aversion behavior [45]. Correlative data from human subjects appear to accord with this role. Subjects with neuropathic pain had decreased blood oxygenation level–dependent activity and gray matter volume in the contralateral thalamus [46]. Furthermore, thalamic connectivity to the cortex is also reduced in patients with chronic pain, which may result in the reduced cortical gray matter [47,48].

A series of important papers on how human thalamic neurons are involved with pain were carried out by Lenz et al. in the 1980s and 1990s [49,50,51,52,53,54]. These included recording neuronal activity in the human principal sensory nucleus of the thalamus and microstimulating ventral thalamic neurons in patients undergoing implantation of DBS electrodes. Microstimulating [55,56] the human thalamic nucleus ventralis caudalis (Vc) in these patients caused acute thermal pain [51]. Neurons in the Vc also respond to specific types of painful stimuli in a graded fashion [50], which provides strong evidence that that pain information is organized somatotopically and by the type of nociceptor.

Even more importantly, some of these recordings were in patients experiencing chronic pain. The thalamic neurons in a patient with post-amputation deafferentation pain demonstrated bursting activity. Bipolar microstimulation of these neurons at 0.3 mA caused a burning pain, similar to the qualitative aspects of patients’ chronic pain, although the location was different [49]. No sensations were describable at currents of 1.0 mA. Brief periods of stimulation with implanted chronic macroelectrodes caused a burning or tingling sensation with effective pain control for at least two years. Comparing thalamic recordings from multiple patients with amputations and control patients who were undergoing DBS for movement disorders showed a much larger thalamic homunculus than the corresponding region in these control patients. The same area also exhibited altered excitability [52]. These results further support the role of the thalamus in modulating excitability in the cortical pain network. Thalamic excitability and organization are related to chronic pain [53].

### 2.3. Insula

The insula has a cytoarchitectonically diverse organization based on posterior (*granular*), intermediate (*dysgranular*), and anterior (*agranular*) subdivisions. It is thought to encode sensation of the physiological condition for the entire body, which is known as interoception [31,57]. Craig et al. [31] showed the existence of 15 distinct cortical areas within the insula of long-tailed macaque monkeys (Macaca fascicularis) [31]. The architectonic map of the macaque insula is an important step towards understanding the connections and function of the insular cortex subareas. Recent models suggest interoceptive afferents are received in the posterior insula, processed by the intermediate insula, and then integrated for efferent autonomic regulation in the anterior insula [58].

A recent functional magnetic resonance imaging (fMRI) study suggested that the anterior insula, skin conductance, and pupil size encode a predictive model of pain. The anterior insula reflected the summed pain expectation and prediction errors from unexpected pain, while the posterior insula encoded pain intensity [59]. In subjects estimating pain intensity, fMRI activity during pain magnitude ratings was matched to that during visual magnitude ratings, to examine how pain perception is an assessment of stimulus intensity [60]. Posterior insular activity correlated with both the magnitude of the visual stimulus used in the task and pain intensity. Modality-specific pain estimation was located further anteriorly. The authors suggested this means pain perception within the insula results from the transformation of nociceptive information into subjective intensity assessment.

The insula is consistently included in circuits whose activity is correlated with pain perception. Some lesions of the insula result in a syndrome called pain asymbolia. In this condition, first described by Schilder in the 1930s, pain perception is intact, yet a patient’s emotional response to pain is inappropriate [61]. Even though both the ACC and insula show elevated activity when viewing painful versus non-painful images [62], the symptoms resulting from insular lesions suggest the insula is more specifically involved in pain empathy. The organization of the insula lends itself to having both affective and somatosensory components. The ACC (discussed next) is likely more involved with affective processing, while the somatosensory cortex is more involved with sensory processing. Widespread damage to the insula, obliterating its function, appears to increase experiential noxious pain perception and somatosensory activation ipsilateral to the lesion [63]. The insula may, therefore, help to suppress pain perception via specific inhibition of the somatosensory cortex.

Interestingly, the insula and the ACC both contain the highest concentrations of von Economo cells in the human brain. These are specialized cells with large cell bodies and axons that project to homeostatic regions in midbrain PAG and the parabrachial nucleus [64]. These cells are thought to mediate rapid social or behavioral inhibition, based on social or cognitive interoception. A recent study using patch clamp recordings of von Economo neurons demonstrated that they are regionally specific excitatory neurons [65]. The loss of these neurons is implicated in many neurological disorders and diseases. The insula and ACC are likely implicated in integrating general interoceptive information, among which pain information is distributed [32].

Direct electrical stimulation of the insula, as in epilepsy mapping, produces a variety of responses, including somatosensory, olfactory, thermal, auditory, and gustatory percepts [66]. Although early investigators, such as Penfield, failed to identify any evoked pain percepts by insular stimulation [33], more recent studies show pain responses from the insula at about 10% of sites. Systematic investigation may clarify the role of the insula in pain processing.

### 2.4. Anterior Cingulate Cortex

The ACC shows activation with pain, anxiety, and cognitive control, suggesting a role in responding to insults that are either corporeal or cognitive [67]. The ACC generally acts as a monitor that signals needed behavioral adjustments in response to corporeal or cognitive challenges [67,68]. In the late 1940s, frontal lobotomies were used to treat intractable pain and addiction with some success [69,70,71]. In the 1960s, Foltz and White [34] reported their experience performing cingulotomies (typically bilaterally) on 16 patients, for whom the affective components of chronic pain were particularly pronounced. The results were poor in only two of the 16 patients, although this was an early study lacking objective pain metrics. Intriguingly, the authors report that signs of opiate withdrawal were also lessened in these patients (14 of whom were “addicted”) after cingulotomy. In 1988, Smith et al. [72] performed surgical cingulotomies on Sprague-Dawley rats, to investigate the role of the cingulate in stress-induced plasma beta-endorphin and morphine withdrawal. Beta-endorphin levels did not significantly increase after cingulotomy or postoperative induced stress, when each was tested independently. However, rat cingulotomy with postoperative induced stress caused a significant increase in plasma beta-endorphin concentrations. These findings suggested cingulum involvement in the regulation of the stress hormone response of beta-endorphin. More so, cingulotomy might be the cause of opiate withdrawal. A series of case reports and case series in humans followed this promising early work. A total of 13 case reports on cingulotomy were published prior to 2008—the majority before 1980 [73]. Results showed variable pain and opiate withdrawal improvements [73,74,75].

In the 1950s, attempts were made to electrically stimulate the ACC to treat chronic pain. Few effects were seen, most of which were adverse—such as speech arrest, vomiting, and tonic muscle contractions [76]. More recent studies using DBS with contemporary electrodes showed improved efficacy and reduced adverse effects [77,78]. For example, in the most extensive study to date using DBS to treat pain, Boccard et al. [79] found a significant 43% improvement on a numeric pain rating scale after six months. Improvements were also seen in other quality-of-life metrics, and on a general health scale. The cognitive and affective elements of chronic pain in these studies showed the most significant improvements, suggesting that ACC DBS may improve these aspects of chronic pain [80].

In comparison, a positive emission tomography (PET) study demonstrated that the ACC is activated during thalamic DBS in patients with chronic pain [55]. Together, these effects strongly implicate the dorsal ACC in processing affective components of pain; however, results about the directionality of cingulate effects are few and mixed. Recent studies have reported the essential nature of examining the temporal dynamics with which the ACC tracks internal cognitive state and induces cognitive control [74,81]. Improved understanding of the temporal dynamics of cingulate function relative to pain may be an important missing piece in understanding the cerebral localization of pain networks.

### 2.5. Beyond the Thalamus, Insula, and ACC

Other recent studies suggested that entirely different brain networks, engaged by the ACC, are responsible for pain regulation. Pain is understood to be a complex experience with sensory, cognitive, and emotional components [82]. Woo et al. [75] indicated that self-regulation of pain and the brain areas responsible for painful experiences are controlled by another circuit. The circuit consists of the genual and subgenual cingulate cortexes, the projections of the nucleus accumbens (NAc, involved in aversion, motivation, and reward valuation), and projections to the ventromedial prefrontal cortex. The suggested involvement of the cingulate is in the valuation of pain or weighting the context of painful stimuli, which preferentially projects to the NAc. A recent rodent study appears to support this mechanism of pain regulation [83]. In this study, the authors suggested that pain relief is associated with learning and motivation to seek environments associated with a relieved state. Endogenous opioid signaling in the rodent cingulate alters dopamine release in the NAc and pain avoidance behavior. Baliki et al. [84] also suggested a role for the NAc in pain valuation and analgesic potential. In this study, NAc activity in response to acute noxious thermal stimuli was compared in control and chronic back pain patients. At the acute noxious stimulus, normal subjects had positive phasic NAc activation, while chronic back pain patients demonstrated negative polarity phasic activity. The authors suggested the onset of acute pain relieved the chronic back pain, which was confirmed psychophysically. Mallory et al. [85] reported on sustained pain relief in a 72-year-old woman with a large right hemisphere infarct, who developed refractory left hemibody pain. Neither the NAc nor the PVG was successful in relieving pain when stimulated in isolation. The combined stimulation reduced the patient’s pain from 10 to 0 at 11 months after surgery. The patient’s post-stroke depression also stayed in remission. These results suggest the emotional aspects of pain can be treated quite well when stimulation involves the NAc.

Such a distributed organization of pain information makes large-scale electrical disruption of gray matter, such as that employed in cingulate DBS or cingulotomy, theoretically unlikely to provide the specificity required to disrupt pain without unintended side effects. These considerations motivate the use of white matter DBS of particular pain-associated tracts, such as the ventral internal capsule, along with the aggregates of grey matter involved in pain processing. It is also important to study the temporal dynamics of pain to determine whether there are temporal or frequency components unique to the development of chronic pain and various pain syndromes. This information may be used to determine when is the best time to modulate information processing in these areas to most effectively improve centralized chronic pain.

## 3. Temporal Dynamics of Pain

Studying the dynamic properties of pain perception is important for disentangling the neural correlates from the described anatomical areas that display a myriad of functions [86]. Few imaging studies have correlated fMRI signals with the temporal properties of pain [86,87,88]. An important consideration in studying the temporal dynamics of pain is that the time constants and activation functions of the various types of peripheral pain fibers are well characterized. The aforementioned unmyelinated C fibers transmit nociceptive information more slowly than myelinated Aδ fibers. These differences in conduction may resonate throughout the system in meaningful ways. As a psychophysical example, the discrimination of different painful sources (i.e., thermal, mechanical, chemical) becomes impossible with the loss of the rapidly conducting myelinated pain fibers. While these peripheral biophysics suggest that temporal dynamics may be necessary for understanding pain perception, studies have yet to use temporal properties of signals to study pain discrimination. It is not clear to what extent, if any, temporal dynamics derived from the biophysics of peripheral sensors apply to chronic pain.

Electroencephalography (EEG) and magnetoencephalography (MEG) are the most readily available noninvasive methodologies to study high-temporal-resolution activity in the brain. EEG has been used to study acute responses to painful stimuli, mostly thermal pain evoked by lasers [89,90,91,92]. Laser-evoked pain has become the dominant psychophysical method for studying pain, because of safety, spatial precision, and how rapidly lasers turn on and off. The source localization of these responses also implicated the dorsal ACC and the insula [93,94]. It is not clear, however, to what extent these studies address the mechanisms underlying chronic pain. Studying chronic pain, per se, is fraught with numerous difficulties. Not only does chronic pain have various causes, but the expression of chronic pain in humans is heterogeneous. It is associated with varied bodily locations, triggers, and unpredictable responses to those triggers. Within chronic pain research, these complications are often simplified in animal models or very specific study populations. This limits the general applicability of results [95].

There have been few and mixed results of EEG studies of chronic pain. A recent meta-analysis of chronic pain EEG studies involved recording subjects during rest, sensory stimulation, and cognitive tasks and showed that subjects with chronic pain have elevated power in various regions across a range of frequencies [96]. Chronic pain was also associated with decreased evoked response amplitudes during cognitive tasks and sensory stimulation [96]. The dominant resting frequencies were typically lower than in healthy controls; however, the specific regions with power and the frequency changes were not consistent across studies. Increased resting frontal theta power was the most reproducible result in these studies. In one study, pathologically high theta power was localized to the insula, although this region is difficult to record with EEG [97]. Theta oscillations are associated with thalamocortical loops, so studies have attributed these oscillations to pathological integration of painful experience into normal circuits. As described, such pathological integration could be due to the regulation of excitability via tonic and burst firing modes in the thalamus. Altered dynamic ranges of frontal theta levels could represent chronic changes in thalamocortical networks.

Several studies have attempted to simulate chronic pain in the laboratory by elongating the duration of painful stimuli. Rhythmic or oscillatory responses to painful stimuli lasting up to tens of minutes in duration are consistently characterized by suppression of alpha- and beta-range power and increases in gamma power [98]. Perturbations of both the bottom-up (i.e., sensory) context and the top-down (i.e., attentional or cognitive) contexts consistently alter pain perception [99,100]. Furthermore, the oscillatory context is important for pain perception, as somatosensory alpha is negatively correlated with pain perception [101,102].

In one notable EEG study, painful tonic stimuli (10-min exposure) correlated with persistent frontocentral gamma power. The source localized to the dorsal ACC [98]. However, the reduction in beta oscillations that other studies have found was more posterior and determined to be correlated with the judgement of stimulus intensity. Such studies using long-duration painful stimuli suggest that the neural representation of pain changes with the duration of the painful experience. Apkarian et al. [103] showed that acute pain stimuli generally activate the somatosensory, insular, and cingulate cortical regions. Patients with chronic back pain had brain activity that localized to the medial prefrontal cortex and into the ACC, which was unique to the chronic pain patients. As the duration of the painful experience increases, fewer somatosensory regions are activated and there is more recruitment from limbic/affective/motivational regions. Their interpretation was that transient nociceptive activity, at some point, is converted into sustained emotional suffering. This result highlights the dynamic nature of chronic pain in time. How these dynamics evolve from the seconds–minutes domain of acute pain to the months and years of chronic pain is unknown, and they are important factors for delineating and treatment.

Finally, intracranial electrophysiological responses to acute painful stimuli in patients implanted with grid electrodes reproduced the changes in delta through beta power described in EEG studies [92,104]. Causal interactions were determined from local field potentials recorded during the response to a painful cutaneous laser stimulus. Cognitive pain control was related to information transfer between the ACC and somatosensory cortices [56,92]. These studies were only carried out in three patients and examined responses to brief thermal pain. Recording broadband high-frequency (e.g., high-gamma; ~70–200 Hz) local field potentials, which correlate with neuronal activity [105,106], yields an intriguing possibility of correlating pain network activity to neuronal populations with high temporal precision. Furthermore, the possibility of altering pain perception with brain stimulation could be highly informative for the development of DBS for chronic pain.

## 4. Pain Illusion Suggests Distinct Roles for the ACC and Insula in Chronic Pain 

**Figure 3:**
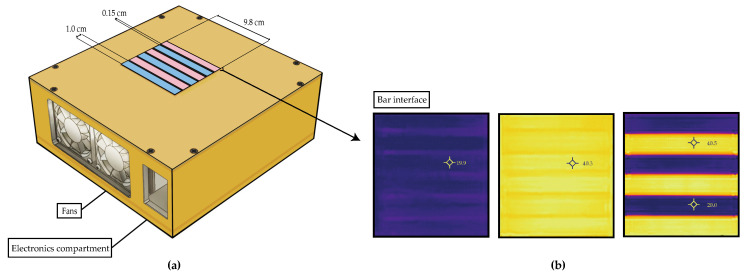
Rendering of a thermal grill interface and infrared images demonstrating patterns of warm and cool temperatures. Drawing (**a**) is representative of the interface that our group constructed. The top of the interface consists of six copper bars. Each bar is 1.0 cm in length and the bars are spaced 0.15 cm apart. The width of the six-bar interface is 9.8 cm. The electronic components fit inside the labeled compartment next to the fans, which allow for necessary air flow. Each bar is connected to a Peltier device, allowing for programmable temperature control (pink and blue coloring represents bars programmed to warm and cool temperatures, respectively). Infrared images acquired using the device are shown in (**b**). Left (**b**) shows all bars set to a cool temperature close to 20.0 °C. Middle (**b**) shows all bars set to a warm temperature close 40.0 °C. Right (**b**) shows one bar set at 20.0 °C and another bar near 40.0 °C. The alternating temperature setting is used to produce the pain illusion.

The “gate control theory” of pain was first described by Melzack and Wall in 1965 [21]; it implicated the relative activation and inhibition of areas in the ascending pain pathway [18]. This theory was sustained in part by studies using the thermal grill illusion (TGI). In the TGI, first discovered by Thunberg in 1896 [107], closely spaced alternating hot and cold stimuli—themselves non-painful—are perceived as painful when felt simultaneously. A three-dimensional rendering of a thermal grill interface constructed by our group is shown in Figure 3a. Each bar on the interface is programmable to a range of temperatures. Images captured with an infrared camera, shown in Figure 3b, demonstrate examples of the temperature arrangements acquired with our thermal grill interface. Since the 1890s, many explanations have been investigated to understand the physiological basis for perceived pain [107,108].

In 1994, Craig and Bushnell suggested that the integration of pain and temperature is the basis of cold-evoked, burning pain [107]. Although the mechanisms remain unclear, the underlying thermal stimuli are thought to inhibit each other, making way for summated nociceptive information without its accompanying somatosensory identity. A recent study in mice showed that the concurrent inhibition and excitation of polymodal channels provides the sensory code for warm perception [108].

Over time, the thermal grill has increased in popularity as a model for studying pain, because it induces neural activity that is perceived as burning pain without actually causing physical harm [109,110]. Clinical studies using the thermal grill have varied from evaluating perceived pain with surveys [111] to using fMRI [112] and PET [113] to evaluate structural involvement. Neuroimaging used in TGI studies suggests that hot or cold stimuli alone activate the insula and somatosensory cortex. Illusory pain from the TGI additionally activates the ACC [113]. The TGI produces a conscious perception of pain, perhaps unique to the ACC. Isolated thermal stimuli (either hot or cold) activate the insula and somatosensory cortex, demonstrating a dissociation between these regions and the cingulate. This further suggests separate roles for the areas involved in pain perception. Bouhassira et al. [114] and others [115,116] have reported that a subset of volunteers in thermal grill experiments are classified as poor or nonresponders. Bouhassira et al. defined these subjects based on not reporting at least one paradoxical painful sensation. There are a variety of reasons someone could be a nonresponder, such as anatomical differences due to calluses, prior injury, or variations in pain tolerance. Including poor-responding subjects in thermal grill experiments during intracranial recording will provide greater understanding of individual variations in pain processing.

Future studies using the thermal grill and neurophysiological data acquired with electroencephalography (ECoG) or stereoelectroencephalography (SEEG) will help to substantiate the temporal dynamics of pain perception. This experimental system may also provide data offering insight on the affective–motivational (“unpleasantness”) and the discriminatory (“pain-intensity”) aspects of pain [112]. Real-time acquisition from the thalamus, insula, and ACC may disentangle the complex relationship between these structures in pain processing. This groundwork will elucidate targets of electrical stimulation as a treatment for chronic pain.

## 5. Conclusions

Pain is a topic of great interest in the medical field because of the current opioid epidemic [6]. Medical providers in the 1990s trained with the establishment of “pain as the 5th vital sign” to improve the quality of patients’ well-being [117]. Increasing pressure to treat pain was met with the availability to prescribe opioids. Although opioids interact with opioid receptors on nerves to block pain signaling, these drugs are problematic for a multitude of reasons, particularly tolerance and lethality. Future pain treatment modalities must be specific to pain but not as problematic for patients’ quality of life as opioids have proven. We believe there is a viable target within the cerebrum based on the history of using DBS to treat pain; however, future work must systematically examine neural structures of interest, in space and time, to gain insight on how these networks interact.

We reviewed the evidence for the functional localization of the conscious perception of pain networks. Many of the gross anatomical correlates of pain are distributed and overlap with areas thought to be involved with interoception, affect, motivation, and cognition. This overlap motivates the need for novel approaches and techniques in understanding the neural mechanisms of pain [118]. The cerebral networks involved in pain are dynamic and distributed. Mapping these intracranial networks will require invasive neurosurgical techniques, such as ECoG and SEEG, to provide high-spatiotemporal-resolution recordings. We suggest that these techniques, in conjunction with the TGI, will provide information on the neurophysiological response of pain. Use of this system also allows for simultaneous qualitative pain evaluation. Further studies on pain intensity in conjunction with neural recordings in the insula, thalamus, and ACC will provide functional considerations for targeted electrical pain therapy in the future.
